# Effect of glucagon-like peptide-1 receptor agonists on heart rate in non-diabetic individuals with overweight or obesity: a systematic review and pairwise and network meta-analysis of randomized controlled trials

**DOI:** 10.1186/s40001-026-03933-9

**Published:** 2026-01-26

**Authors:** Yiwen Zhang, Chuying Zhang, Xuyang Gong, Panpan Cheng, Hang Fu, Linqi Diao, Chunhua Song

**Affiliations:** 1 Department of Medical Technology, Hongyi College, Henan Medical University, Xinxiang, 453003 Henan China; 2 Department of Epidemiology and Statistics, College of Public Health, Zhengzhou University, Zhengzhou, 450001 Henan China; 3 Division of Endocrinology, Department of Internal Medicine, The First Affiliated Hospital of Zhengzhou University, Zhengzhou, 450052 Henan China; 4 Division of Respirology, Department of Internal Medicine, The First Affiliated Hospital of Zhengzhou University, Zhengzhou, 450052 Henan China; 5 Institute for Hospital Management of Henan Province, Zhengzhou, 450000 Henan China; 6 The First Affiliated Hospital of Zhengzhou University, Zhengzhou, 450052 Henan China; 7 Henan Provincial Center for Disease Control and Prevention, Zhengzhou, 450052 Henan China

**Keywords:** Glucagon-like peptide-1 receptor agonists, Heart rate, Obesity, Adverse (side) effects, Systematic review, Network meta-analysis

## Abstract

**Objectives:**

To explore the association of glucagon-like peptide-1 receptor agonists (GLP-1RAs) on heart rate (HR) in overweight or obese patients without diabetes.

**Methods:**

A comprehensive search of the PubMed, Web of Science, Embase, and Cochrane Library databases was conducted. Mean differences (MDs) were calculated as effect estimates for HR. Pairwise and network meta-analysis were conducted.

**Results:**

Twelve articles were included. Pairwise meta-analysis showed significant association of increase compared with placebo in liraglutide [MD 2.37, 95% confidence interval (CI) 1.86, 2.89], semaglutide (MD = 3.35; 95% CI 1.69, 5.01), orforglipron (MD = 7.30; 95% CI 5.48, 9.12), oral semaglutide (MD = 4.50; 95% CI 3.11, 5.89), tirzepatide (MD = 2.05; 95% CI 0.96, 3.13), retatrutide (MD = 3.46; 95% CI 1.74, 5.18), and total GLP-1RAs (MD = 3.47; 95% CI 2.65, 4.29). Network meta-analysis revealed that orforglipron 36 mg was associated with the most pronounced increase (MD = 9.29; 95% CI 4.45, 13.86), whereas tirzepatide 5 mg was associated with the least increase (MD = 0.52; 95% CI − 2.71, 3.78).

**Conclusions:**

GLP-1RAs were associated with the increasing of HR in patients with overweight or obesity. Orforglipron 36 mg was associated with the most pronounced increase, and tirzepatide 5 mg the least.

**Supplementary Information:**

The online version contains supplementary material available at 10.1186/s40001-026-03933-9.

## Introduction

Obesity is a critical global public-health concern as it is associated with a broad spectrum of serious comorbidities, including diabetes, hypertension, dyslipidemia, cardiovascular disease, and cancer [1, 2]. Over the past few decades, the global prevalence of obesity has gradually increased, and this trend is projected to persist [3]. Dietary and exercise control are insufficient to address the obesity epidemic [4, 5], prompting the development of a wide range of pharmacotherapies for weight management [6, 7]. Among these agents, glucagon-like peptide-1 receptor agonists (GLP-1RAs) have emerged as the particularly promising class, exhibiting robust weight loss effects [8, 9].

However, the marked therapeutic benefits of GLP-1RAs are often accompanied by significant adverse effects that may lead to treatment discontinuation and, in some cases, serious clinical consequences [10]. Thus, a comprehensive understanding of these side effects is essential to facilitate precise medicine in obesity management. Previous work has indicated that the use of GLP-1RAs in Type 2 diabetes mellitus (T2DM) was usually accompanied by an increased heart rate (HR) [11, 12]. Elevated resting HR is a well-established risk factor for adverse cardiovascular events [13]. In addition, a negative correlation has been observed between HR and survival in the general population and patients with cardiovascular disease [14]. While several investigations have evaluated the effects of GLP-1RAs on HR, few have focused specifically on populations with obesity. These studies are also limited to a single drug and lack systematic synthesis of HR data or direct comparisons among different GLP-1RAs [15, 16]. Meanwhile, although GLP-1RAs could significantly increase HR, some evidence shows that liraglutide (LIR) does not raise HR during acute myocardial infarction [17, 18]. Overall, evidence regarding the impact of GLP-1RAs on HR in patients with obesity remains limited and inconsistent. Nevertheless, clarifying the magnitude and variability of HR effects across different GLP-1RAs is of paramount importance, as it enables accurate medication selection for patients who cannot tolerate HR elevation.

In the present study, we performed paired and network meta-analyses to thoroughly evaluate the impact of GLP-1RAs on HR in patients with obesity by comprehensively searching and synthesizing the latest clinical trial evidence. The findings of this study were intended to provide critical evidence for the rational use of GLP-1RAs and inform clinical decision-making in obesity management.

## Materials and methods

Pairwise and network meta-analysis were conducted in adherence to the Preferred Reporting Items for Systematic Reviews and Meta-Analyses (PRISMA) guidelines [19, 20] and prospectively registered (PROSPERO ID: CRD42024537690).

### Search strategy and study selection

Publications were retrieved from PubMed, Embase, Web of Science, and the Cochrane Library from database inception to July 31, 2025. In addition, manual searches of relevant journals and ClinicalTrials.gov were conducted within the same time frame. The databases were searched using the following Medical Subject Headings (MeSH) terms or keywords: (1) glucagon-like peptide-1 receptor agonist AND (2) randomized OR controlled trial randomized AND (3) overweight or obesity. Two independent reviewers (Z.Y.W and Z.C.Y) searched the databases and screened the titles, abstracts, and full-text articles to select the studies. Discrepancies during the screening process were resolved through consultation with other members of the research team. The complete search strategy is detailed in Table S1.

### Inclusion and exclusion criteria

Studies that reported data on HR in research on GLP-1RAs were included. The study population was restricted to overweight or obese patients without diabetes. We included only randomized controlled trials (RCTs) of at least 26 weeks that compared GLP-1RAs with another GLP-1RAs or placebo. Studies were excluded if they involved patients of age under 18 years, with a history of renal, hepatic failures, or other serious diseases. Moreover, phase I studies, secondary analyses, and unpublished data were not used. Two independent reviewers (Z.Y.W and Z.C.Y) evaluated the eligibility of studies for inclusion in the analysis. Disagreements were discussed with other team members until consensus was reached to include only those articles that best met the criteria.

### Outcome definitions

The only outcome of this systematic review and meta-analysis was the change in HR from baseline to the study endpoint.

### Quality assessment

The Cochrane Risk of Bias Tool, version 2, for randomized trials was used to assess the overall risk of bias [21]. Trials with all domains at low risk of bias were considered to have an overall low risk of bias, whereas trials with at least one domain at high risk of bias were considered to have an overall high risk of bias. Quality assessment was performed by two independent reviewers (C.P.P. and G.X.Y), and disagreements were resolved by consensus.

### Data extraction

Data extraction was performed by two independent reviewers (Z.Y.W and Z.C.Y), and the extracted content included the following: study details including author(s), publication year, study design, sample size; population characteristics including patients, age, gender, body weight, body mass index, waist circumference, baseline HR, and inclusion/exclusion criteria; intervention and comparison including types of GLP-1RAs, oral or injectable medications; and outcome indicators, primarily changes in HR from baseline.

### Data synthesis

The mean and standard deviation (SD) of baseline and post-intervention HR were extracted from each study for the intervention and control groups. Some trials reported multiple time-point results, but only the most recent time point was extracted for analysis. When the SD of HR variability was not provided directly, the SD of the mean group was calculated using the formulas in the Cochrane Handbook chapter based on the standard error of the mean (SEM) or 95% CI (SD = SEM * $\sqrt{{\boldsymbol{n}}}$ or SD = $\sqrt{{\boldsymbol{n}}}$* (upper limit − lower limit)/3.92) [22]. When not reported, change from baseline SDs were estimated using the equation from the Cochrane Handbook chapter, assuming a correlation coefficient of 0.50 between baseline and post-intervention HR ($\sqrt{{SD}_{baseline}^{2}+{SD}_{final}^{2}-2*0.5*{SD}_{baseline}{* SD}_{final}}$) [23, 24].

Conventional pairwise meta-analyses were pooled using a random-effect model comparing means with their SDs. The Higgins I^2^ was used to assess statistical heterogeneity between studies; an I^2^ statistic of > 50% was considered significant heterogeneity, and a value of < 50% was considered acceptable. Subgroup analyses were performed based on different medications and population characteristics. In all cases, a two-tailed *p* value < 0.05 was deemed statistically significant for all analyses.

A network meta-analysis was conducted using Bayesian methods to evaluate the relative effects of treatment on HR for each study group. This approach synthesizes all available evidence, including both direct and indirect comparisons, when a connected evidence network exists. Point estimates and 95% confidence intervals (CIs) were assessed using the Markov chain Monte Carlo method with a random-effect model, and 95% CIs excluding 0 were considered significant. A network plot of all interventions was made to identify possible direct and indirect comparisons. Ranking probabilities for all inventions were estimated using the surface under the cumulative ranking curve (SUCRA) and mean ranks to rank the effect of each invention. Forest plots (versus placebo) and league tables were generated to visually present the results of the network meta-analysis [25]. Subgroup analyses were conducted to explore potential sources of heterogeneity in HR responses to GLP-1RAs. These exploratory analyses included stratification by formulation, dosing frequency, number of activated channels, treatment duration, age and baseline heart rate. Publication bias was assessed by visual inspection of the Begg’s funnel plots and Egger’s asymmetry test. Sensitivity analyses were conducted by comparing fixed effects with random effects and randomly deleting a reference to assess the stability of the result. A *p* value greater than 0.05 indicated that inconsistency was not evident.

All statistical analyses in this study were performed in this study using R version 4.5.2 (http://www.r-project.org/) with the BUGSnet, netmeta, and gemtc packages [26, 27].

## Results

### Study selection and characteristics

**Fig. 1:**
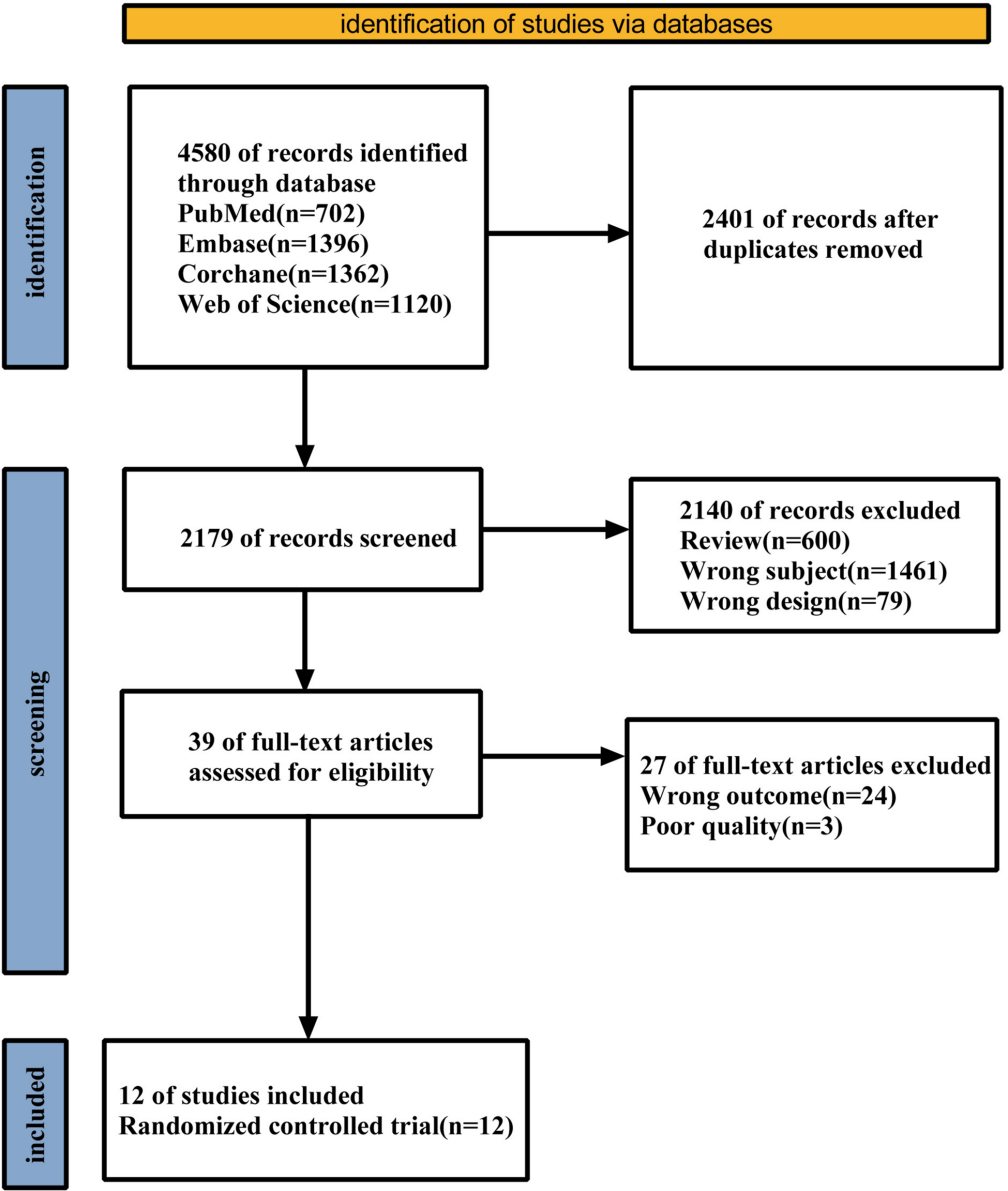
PRISMA flowchart of the included studies.

The PRISMA flowchart of the included studies is shown in Fig. 1. A total of 4580 studies were initially retrieved from four databases. After excluding studies that did not meet the criteria or were repeated, 12 RCTs were included. Among them, 10 were phase 3 controlled clinical trials and 2 were phase 2 controlled trials. The study duration was 36–160 weeks. Three studies were treated with semaglutide (SME) [28–30], three with LIR [31–33], one with SME or LIR [34], one with oral semaglutide (OSM) [35], one with orforglipron (ORF) [36], two with tirzepatide (TZP) [37, 38] and one with retatrutide (RET) [39]. A total of 15 313 patients were included, 9 128 in the GLP-1RAs group and 6 185 in the control group. The mean baseline HR was observed to range from 69.0 bpm to 74.0 bpm, and the changes in HR from baseline exhibited a range of 0.6 bpm to 7.4 bpm. The detailed characteristics of the included studies are shown in Table 1. In addition, concomitant medications were permitted to be used in all studies and the detailed medication requirements in the experiment are shown in Table S2.

**Table 1:** Characteristics of included studies

Study, First author year	ClinicalTrials.gov Identifier	Study phase	Study arm	Sample size	Study duration, weeks	Age, years	Male, %	BMI, kg/m^2^	Waist circumference, cm	Baseline HR, bpm Mean ± SD	HR change from basine, bpm Mean ± SD
Louis 2024	NCT04660643	III	TZP 10 or 15 mg	335	88	49	99(30)	38.4	107.3 ± 22.3	72.0 ± 9.0	3.2 ± 8.29
			Placebo	335		48	98(30)				0.1 ± 8.29
Sean 2023	NCT05051579	II	ORF 12 mg	50	36	49.8	19(38)	37.7	114.4 ± 16.5	73.9 ± 9.1	5.3 ± 9.42
			ORF 24 mg	53		57	23(43)	38.1	120.1 ± 19.1	71.9 ± 12.1	3.2 ± 9.46
			ORF 36 mg	58		55.9	11(38)	38	117.3 ± 15.5	69.4 ± 9.3	7.4 ± 9.52
			ORF 45 mg	61		53.7	26(43)	37.8	116.9 ± 13.8	69.8 ± 10.0	6.0 ± 9.61
			Placebo	50		54	21(42)	37.8	115.5 ± 15.4	69.6 ± 10.6	− 1.8 ± 9.48
Filip 2023	NCT05035095	III	OSM 50 mg	334	68	49	87(26)	37.3	112.6 ± 13.6	71.0 ± 10.0	4.1 ± 9.14
			Placebo	333		50	95(29)	37.7	114.5 ± 15.4	72.0 ± 10.0	− 0.4 ± 9.12
Ania 2023	NCT04881760	II	RET 1 mg	69	48	50.6	36(52)	37.5	114.8 ± 14.7	71.1 ± 11.2	1.7 ± 9.96
			RET 4 mg	67		48.8	35(52)	37.4	116.2 ± 15.4	69.9 ± 10.7	3.1 ± 6.34
			RET 8 mg	70		47.4	36(51)	37.2	114.5 ± 13.5	70.6 ± 10.1	4.8 ± 8.76
			RET 12 mg	62		45.8	32(52)	37.4	116.5 ± 16.4	69.3 ± 9.2	6.0 ± 9.24
			Placebo	70		48	36(51)	37.3	115.1 ± 13.9	74.0 ± 10.7	0.4 ± 8.11
Domenica 2022	NCT04074161	III	SME 2.4 mg	126	68	48	24 (19)	37	111.8 ± 16.3	71.0 ± 9.0	5.4 ± 9.74
			LIR 3.0 mg	127		49	30 (24)	37.2	113.5 ± 15.0	71.0 ± 10.0	4.3 ± 10.06
			Placebo	85		51	19 (22)	38.8	115.4 ± 15.1	72.0 ± 10.0	1.2 ± 9.64
Ania 2022	NCT04184622	III	TZP 5 mg	630	72	45.6	204(32)	37.4	113.2 ± 14.3	72.3 ± 9.6	0.6 ± 8.78
			TZP 10 mg	636		44.7	209(33)	38.2	114.8 ± 15.8	71.8 ± 9.6	2.3 ± 8.83
			TZP 15 mg	630		44.9	205(33)	38.1	114.4 ± 15.6	72.5 ± 10.0	2.6 ± 8.78
			Placebo	643		44.4	207(32)	38.2	114.0 ± 14.9	72.9 ± 9.3	0.1 ± 9.38
Timothy 2022	NCT03693430	III	SME 2.4 mg	152	104	47.3	29 (19)	38.6	115.8 ± 14.3	73.0 ± 11.0	3.3 ± 10.15
			Placebo	152		47.4	39 (26)	38.5	115.7 ± 15.5	72.0 ± 9.0	− 0.8 ± 9.54
John 2021	NCT03548935	III	SME 2.4 mg	1306	68	46	351(27)	37.8	114.6 ± 14.8	72.0 ± 10.0	3.5 ± 9.54
			Placebo	655		47	156(24)	38	114.8 ± 14.4	72.0 ± 10.0	− 0.7 ± 10.00
Thomas 2021	NCT03611582	III	SME 2.4 mg	407	68	46	92 (23)	38.1	113.6 ± 15.1	71.0 ± 10.0	3.1 ± 10.00
			Placebo	204		46	24 (12)	37.8	111.8 ± 16.2	71.0 ± 10.0	2.1 ± 10.00
Julie 2021	NCT04122716	III	LIR 3.0 mg	49	52	43	–	32.6	100.3 ± 10.0	69.0 ± 12.0	4.4 ± 10.36
			Placebo	49						–	− 0.9 ± 10.36
Carel 2017	NCT01272219	III	LIR 3.0 mg	1472	160	47.5	364(24)	38.8	116.5 ± 14.4	–	2.1 ± 10.00
			Placebo	738		47.3	176(23)	39	116.7 ± 13.9	–	− 0.02 ± 9.80
Xavier 2015	NCT01272219	III	LIR 3.0 mg	2487	56	45.2	530(21)	38.3	115.0 ± 14.4	–	2.5 ± 9.80
			Placebo	1224		45	273(22)	38.3	114.5 ± 14.3	–	0.1 ± 9.50

BMI: body mass index; HR: heart rate; bpm: beats per minute; SD: standard deviation; TZP: tirzepatide; ORF: orforglipron; OSM: oral semaglutide; RET: retatrutide; SME: semaglutide; LIR: liraglutide

### Risk-of-bias analysis

The risk of bias of the included RCTs is presented in Figure S1. Among these RCTs, three studies may have a certain impact on outcome events due to loss to follow-up, two may not have been double blind, and the other biases were low in all studies. In general, these studies had a low or moderate level of risk.

### Pairwise meta-analysis

**Fig. 2:**
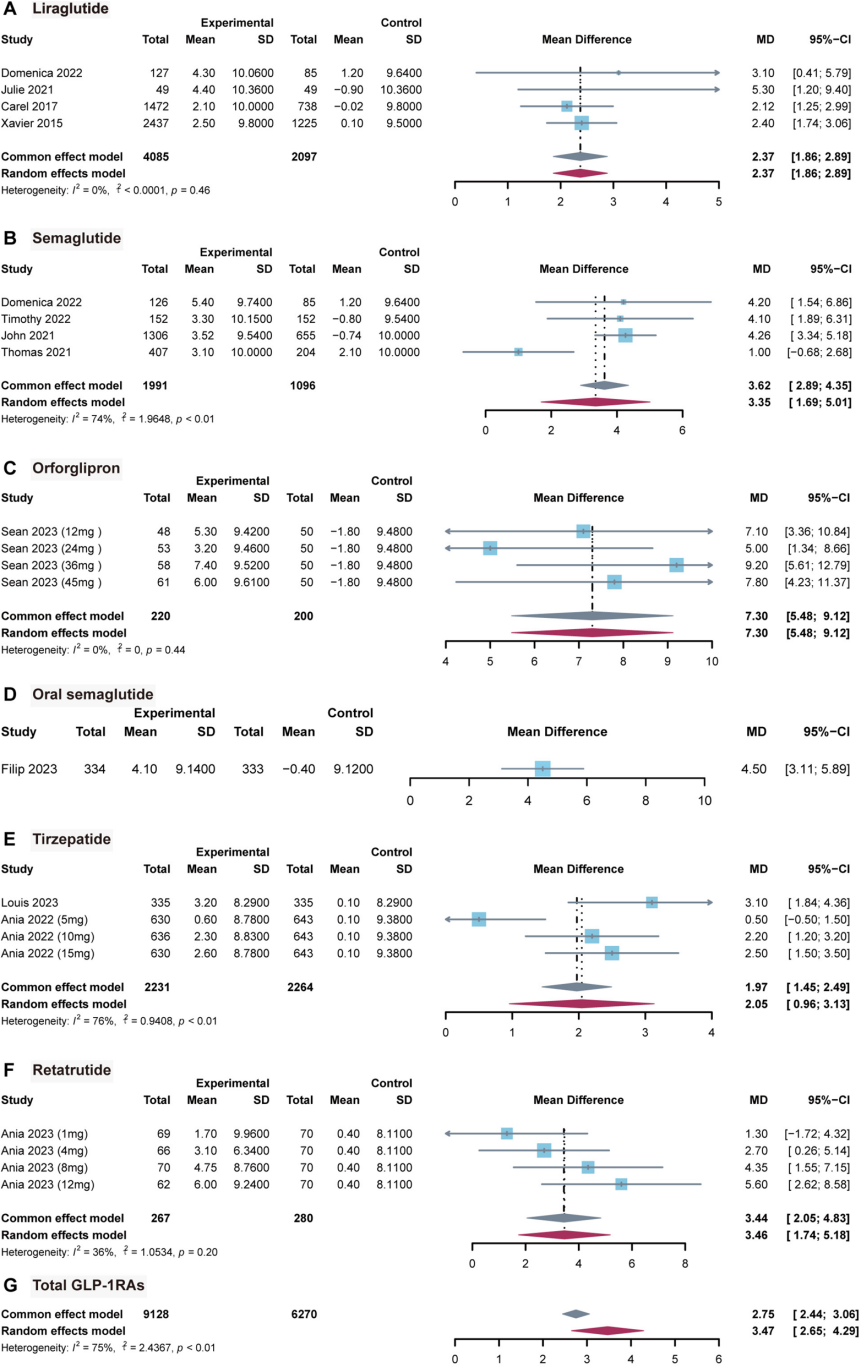
Forest plots for pairwise meta-analysis. GLP-1 RAs: glucagon-like peptide-1 receptor agonists; MD: mean difference

Twelve articles were included in the pairwise meta-analysis. Six study were summarized from original research [LIR, SME, ORF, OSM, TZP, and RET]. Overall, the results indicated a statistically significant association between GLP-1RAs and increased HR when compared with placebo [median deviation, (MD) = 3.47; 95% CI 2.65, 4.29; Fig. 2G]. Specifically, a statistically significant association between LIR use and elevated HR was observed with a relatively smaller magnitude (MD = 2.37; 95% CI 1.86, 2.89; Fig. 2A), as well as similar associations in SME (MD = 3.35; 95% CI 1.69, 5.01; Fig. 2B) and TZP (MD = 2.05; 95% CI 0.96, 3.13; Fig. 2E). For ORF and RET, pronounced statistically significant associations with greater HR elevation were identified (MD = 7.30; 95% CI 5.48, 9.12, Fig. 2C; MD = 3.46; 95% CI 1.74, 5.18; Fig. 2F). In addition, one study reported HR data for OSM with a combined association indicating elevated HR (MD = 4.50; 95% CI 3.11, 5.89; Fig. 2D).

### Network meta-analysis

**Fig. 3:**
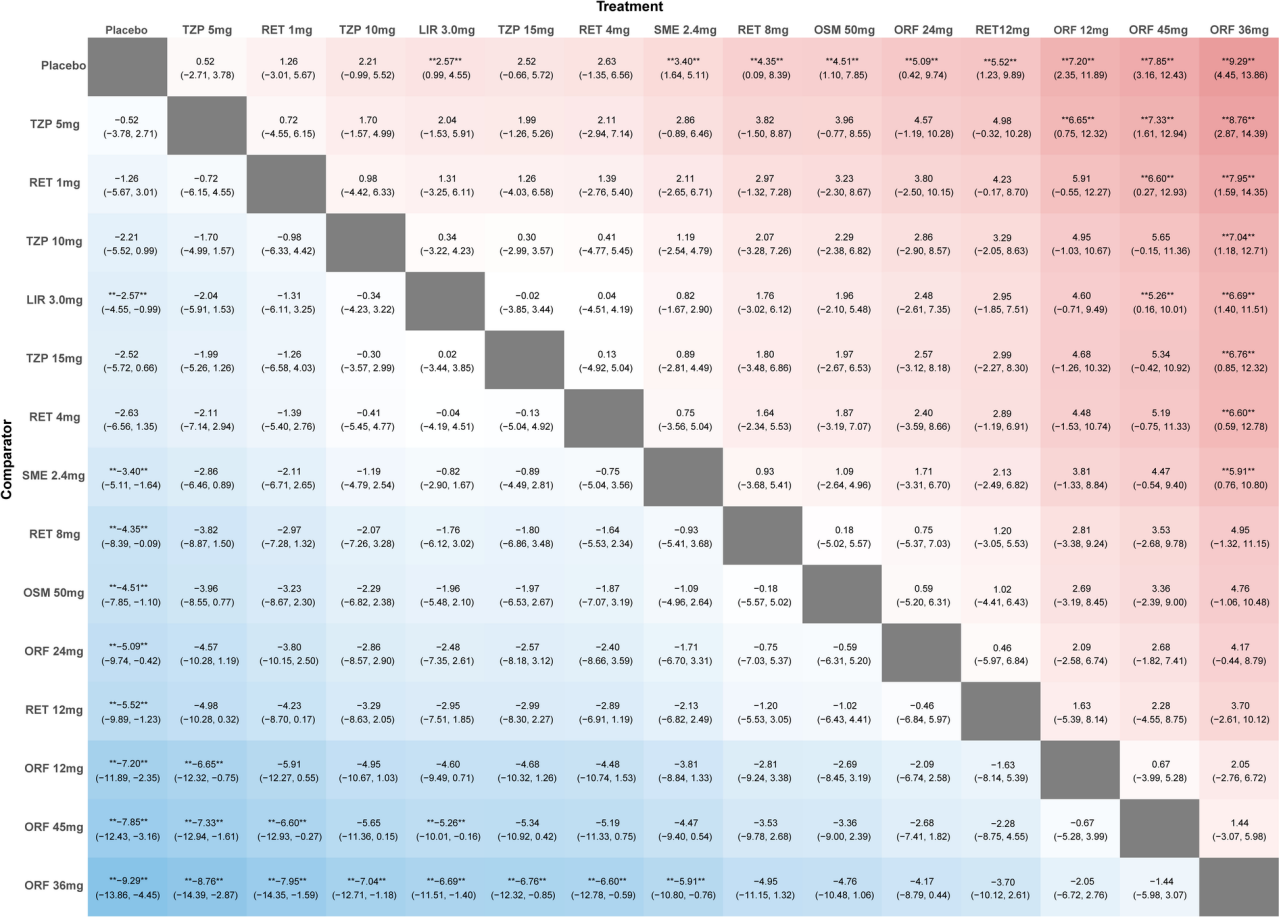
League table for network meta-analysis. TZP: tirzepatide; ORF: orforglipron; OSM: oral semaglutide; RET: retatrutide; SME: semaglutide; LIR: liraglutide

Eleven studies were included in this network meta-analysis (one study was excluded because of unclear TZP dosage). Network plots of all outcomes are illustrated in Figure S2. Compared with placebo, TZP 5 mg (MD = 0.52; 95% CI − 2.71, 3.78), TZP 10 mg (MD = 2.21; 95% CI − 0.99, 5.52), TZP 15 mg (MD = 2.52; 95% CI − 0.66, 5.72), SME 2.4 mg (MD = 3.40; 95% CI 1.64, 5.11), LIR 3.0 mg (MD = 2.57; 95% CI 0.99, 4.55), RET 1 mg (MD = 1.26; 95% CI − 3.01, 5.67), and RET 4 mg (MD = 2.63; 95% CI − 1.35, 6.56) were associated with weaker magnitude of HR elevation. In addition, RET 8 mg (MD = 4.35; 95% CI 0.09, 8.39), RET 12 mg (MD = 5.52; 95% CI 1.23, 9.89), OSM 50 mg (MD = 4.51; 95% CI 1.10, 7.85), ORF 12 mg (MD = 7.20; 95% CI 2.35, 11.89), ORF 24 mg (MD = 5.09; 95% CI 0.42, 9.74), ORF 36 mg (MD = 9.29; 95% CI 4.45, 13.86), and ORF 45 mg (MD = 7.85; 95% CI 3.16, 12.43) showed more pronounced associations with HR elevation. More detailed information can be found in Figs. 3 and S3. When all drugs were ranked based on their observed associations with HR, ORF 36 mg was linked to the greatest magnitude of HR increasing and TZP 5 mg was associated the minimum. The detailed SUCRA is in Figure S4, and no evidence of inconsistency was found in this network analysis (Figure S5).

### Subgroup analysis

We conducted subgroup analyses to explore potential associations between different medications, population characteristics and HR changes. Exploratory findings suggested that oral medication may be associated with a greater increase compared to hypodermic injection medication (Figure S6). Similarly, medication once a week may be linked to greater relative to medication once a day (Figure S7). We observed that single-channel activation was associated with a greater increase in heart rate (Figure S8). Furthermore, results indicated potential relationships that longer treatment duration, younger patient age, and lower baseline HR were each linked to a smaller magnitude of HR elevation (Figures S9–11).

### Sensitivity analysis and publication bias

For each incorporation effect, we simultaneously applied fixed effects, and the results (Fig. 2) were similar to random effects. We also randomly deleted one study, and the results did not change significantly (Figure S12). These findings indicated that our results were stable. Funnel plots of pairwise and network meta-analyses were visually symmetrical (Figures S13, 14). Egger's test showed the total GLP-1RAs group may have some publication bias (Table S3).

## Discussion

This pairwise and network meta-analysis comprehensively evaluated and compared the effect of six GLP-1RAs on HR in obese adults, encompassing three novel drugs (ORF, RET, and TZP). Our pooled data analysis, based on high-confidence estimates, demonstrated that all GLP-1RAs were significantly associated with elevated HR. In terms of the associations between individual GLP-1RAs and HR elevation, ORF was linked to the most magnitude of increasing HR, whereas TZP was associated with the least. A potential dose-dependent pattern was apparent. The 36 mg dose of ORF was correlated with the most pronounced HR increase and the 5 mg dose of TZP was linked to the minimum. Furthermore, our findings suggested that oral and long-acting GLP-1RAs may be associated with a more substantial increase. In addition, a trend was identified, wherein the association between heart-rate-increasing and these drugs diminished over the course of treatment.

The precise mechanism underlying GLP-1RA-induced HR elevation remains incompletely understood, though several potential explanations have been proposed. The GLP-1 receptor is directly expressed in human cardiac sinus node cells, and GLP-1RAs activate this receptor to mediate an increased HR [40, 41]. This is corroborated by clinical studies conducted on patients with T2DM [42]. Nevertheless, the isolated atrial GLP-1R does not exert a direct chronotropic effect upon exposure to GLP-1RAs in intact hearts [43]. This finding indicates that the rise in HR is at least partially attributable to an increase in central or local sympathetic nervous system activity or to a reduction in parasympathetic nervous system inputs [44]. Recent studies show that GLP-1RAs may regulate HR via preproglucagon neurons in the nucleus tractus solitarius [45]. This increase in HR has also been previously postulated to be a compensatory response to the decrease in blood pressure caused by vasodilation induced by GLP-1 [41]. However, in a head-to-head study, no significant correlation was found between blood pressure and HR [46]. GLP-1RAs have been demonstrated as well to decrease HR variability while simultaneously increasing HR [11]. Interestingly, although a substantial body of evidence indicates that elevated HR is an independent risk factor for cardiovascular events and mortality [13], a number of clinical studies and meta-analyses have demonstrated that the utilization of GLP-1RAs is associated with reduced incidence of cardiovascular events [47, 48]. At present, the cardioprotective effect of GLP-1RAs after synthesis through multiple pathways is indisputable, which may be related to the powerful weight loss, antihypertensive, anti-inflammatory, and other metabolism-improving effects of GLP-1RAs. Conversely, their direct effect on the heart remains unclear and requires further study. Meanwhile, the gastrointestinal adverse effects of GLP-1 progressively decrease with the duration of drug administration. In this study, the trend was the same as the effect of increasing HR, which may be related to a decrease in the sensitivity of the corresponding receptor [10, 49].

To the best of our knowledge, this study is the first systematic review and network meta-analysis to evaluate the effect of HR in obese adults. Previous meta-analyses have either focused on a limited number of GLP-1RAs and lacked comprehensive comparisons across agents [11, 15, 42]. In contrast, this study provides the most extensive and up-to-date data to support evidence-based clinical decision-making, particularly for patients unable to tolerate HR elevation. This study is comparable to a net meta-analysis conducted with T2DM 10 years ago, which concluded that GLP-1RAs increase HR [18]. Another research has provided reliable evidence of TZP in T2DM, but it includes only one drug and lacks comparisons with other GLP-1RAs [50]. Most studies have primarily investigated the effect on HR but not the factors that may influence this effect. The article investigated exenatide and LIR and concluded that these two increase the HR [51]. They also found that longer-acting medications may cause a more pronounced increase in HR [42].

Although the increase in HR caused by most GLP-1RAs agents is relatively small, its clinical significance varies among different populations. For high-risk groups, including patients with existing cardiovascular damage, such as heart failure, arrhythmia, or coronary artery disease, as well as adults aged 65 or older, even this minor increase in HR may have clinical significance. It is well-known that aging impairs the regulation of HR by the autonomic nervous system and increases sensitivity to hemodynamic changes [52]. It is noteworthy that in the group of patients with heart failure, per 5 bpm higher HR is associated with worse prognosis [53]. This study provides detailed data related to HR, which can guide personalized medication. For high-risk patients, if the GLP-1RAs used can reduce the magnitude of the increase in heart rate, then while retaining the benefits of GLP-1RAs in metabolism and cardiovascular aspects, minimizing the burden related to heart rate may be more beneficial.

Our work had several strengths. It incorporated almost all GLP-1RA drugs for the treatment of obesity. The effects of different GLP-1RAs, different dosages, and characteristics of the drug or the patients were also assessed using scientific statistical methods, allowing for a comprehensive evaluation. Our inclusion of only high-quality RCTs lent credibility to the results, and sensitivity analyses demonstrated the robustness of our findings.

Nevertheless, this study also had some limitations. First, our analysis is the potential inclusion of participants with prediabetes. This raises the possibility that the observed associations between GLP-1RAs and HR elevation may be partially confounded by underlying prediabetes status. Second, factors such as background medication and exercise that may influence HR were not considered when combining effects. Third, the random-effect model was inadequate for addressing the substantial heterogeneity observed in some of the included studies, which diminished the credibility of the results. In addition, the heterogeneity of the baseline characteristics of each trial included in this study may indirectly affect the results. Furthermore, after excluding one study with the largest difference in outcome, the heterogeneity of the associations between semaglutide and HR elevation (MD = 4.23; 95% CI 3.42, 5.05; *I*^2^ = 0%; Figure S15) and between tirzepatide and HR elevation (MD = 2.53; 95% CI 1.91, 3.15; *I*^2^ = 0%; Figure S16) was significantly reduced. This observation suggests a potential link between the previously observed heterogeneity in the tirzepatide subgroup and variations in dosage. Fourth, some trials were at potential risk of bias, including open-label design and pharmaceutical industry funding. In addition, publication bias may overestimate the true magnitude of GLP-1RA-induced HR elevation. Finally, strict inclusion criteria to ensure literature quality resulted in a relatively small number of studies for some agents, with certain GLP-1RAs evaluated in only a single trial, introducing potential randomization bias. As additional GLP-1RA clinical trials are conducted, we plan to update our analyses to enhance result reliability.

In conclusion, this study suggested significant associations between GLP-1RAs and elevated HR in obese patients versus placebo. ORF 36 mg links to the most pronounced HR elevation, and TZP 5 mg to the least. Treatment duration, baseline HR, and other factors may relate to HR elevation variability. Our findings inform GLP-1RA clinical consideration in obese patients, especially those with cardiovascular comorbidities or HR intolerance, and aid formulation selection. Further studies are needed to explore GLP-1RA–HR association mechanisms and broader cardiovascular relationships. Future research should include larger, well-powered trials for newer agents and publication of negative or neutral findings to reduce publication bias and enhance the robustness of conclusions.

## Supplementary Information

Supplementary material 1.

## Data Availability

The study-specific summary data included in the meta-analysis can be obtained from the corresponding authors.
