# Supplementary material for: Effect of glucagon-like peptide-1 receptor agonists on heart rate in non-diabetic individuals with overweight or obesity: a systematic review and pairwise and network meta-analysis of randomized controlled trials
Source: Eur J Med Res. 2026 Jan 26;31:318. doi: 10.1186/s40001-026-03933-9 (PMC12918571; doi:10.1186/s40001-026-03933-9)
Supplement: Supplementary file 1 — Supplementary material 1. [file 40001_2026_3933_MOESM1_ESM.docx]

**Supplementary Material**

**Effect of glucagon-like peptide-1 receptor agonists on heart rate in non-diabetic individuals with overweight or obesity: A systematic review and pairwise and network meta-analysis**

**Contents**

**Supplementary Table 1** Data sources and search strategies in the meta-analysis.

**Supplementary Table 2** Other drugs used during the experiment.

**Supplementary Table 3** Results of the Egger test.

**Supplementary Figure 1** Assessment of the risk of bias in the included studies.

**Supplementary Figure 2** Network plots of included GLP-1 RAs.

**Supplementary Figure 3** Forest plots of network meta-analysis.

**Supplementary Figure 4** SUCRA plots of network meta-analysis.

**Supplementary Figure 5** Consistency of network meta-analysis.

**Supplementary Figure 6** Subgroup analysis of oral or injection medication.

**Supplementary Figure 7** Subgroup analysis of medication interval.

**Supplementary Figure 8** Subgroup analysis of activated channels.

**Supplementary Figure 9** Subgroup analysis of treat time.

**Supplementary Figure 10** Subgroup analysis of age.

**Supplementary Figure 11** Subgroup analysis of baseline heart rate.

**Supplementary Figure 12** Sensitivity analysis of randomly deleting one study.

**Supplementary Figure 13** Funnel plots of pairwise meta-analyses.

**Supplementary Figure 14** Funnel plots of network meta-analyses.

**Supplementary Figure 15** Forest of semaglutide after excluding one study.

**Supplementary Figure 16** Forest of tirzepatide after excluding one study.

**Supplementary Table 1** Data sources and search strategies in the meta-analysis.

| **Databases** | **Search number** | **Query** | **Results** |
| --- | --- | --- | --- |
| **PubMed**  **732** | #1 | (obesity[Title/Abstract]) OR (overweight [Title/Abstract]) OR (obese [Title/Abstract]) | 421020 |
|  | #2 | (Glucagon Like Peptide 1 Receptor Agonists [Title/Abstract]) OR (glucagon-like peptide-1 receptor agonist [Title/Abstract]) OR (Retatrutide [Title/Abstract]) OR (Tirzepatide [Title/Abstract]) OR (Mazdutide [Title/Abstract]) OR (CagriSema [Title/Abstract]) OR (semaglutide [Title/Abstract]) OR (Orforglipron [Title/Abstract]) OR (dulaglutide [Title/Abstract]) OR (liraglutide [Title/Abstract]) OR (exenatide [Title/Abstract]) OR (beinaglutide [Title/Abstract]) OR (Albiglutide [Title/Abstract]) OR (Lixisenatide [Title/Abstract]) OR (loxenatide [Title/Abstract]) | 1297432 |
|  | #3 | randomized controlled trial[Publication Type] OR randomized[Title/Abstract] OR placebo[Title/Abstract] OR randomly [Title/Abstract] | 1308444 |
|  | #4 | #1 AND #2 AND #3 | 732 |
| **Web of science 1120** | #1 | TS=(obesity OR overweight OR obese ) | 565902 |
|  | #2 | TS=(Glucagon Like Peptide 1 Receptor Agonists OR glucagon-like peptide-1 receptor agonist OR Retatrutide OR Tirzepatide OR Mazdutide OR CagriSema OR semaglutide OR Orforglipron OR dulaglutide OR liraglutide OR exenatide OR beinaglutide OR Albiglutide OR Lixisenatide OR Loxenatide ) | 17558 |
|  | #3 | TS=(randomized controlled trial OR randomized OR placebo OR randomly ) | 1622144 |
|  | #4 | #1 AND #2 AND #3 | 1120 |
| **Embase 1396** | #1 | 'obesity ':ab,ti OR 'overweight ':ab,ti OR 'obese':ab,ti | 610777 |
|  | #2 | 'Glucagon Like Peptide 1 Receptor Agonists':ab,ti OR 'glucagon-like peptide-1 receptor agonist':ab,ti OR 'Retatrutide':ab,ti OR 'Tirzepatide':ab,ti OR 'Mazdutide':ab,ti OR 'CagriSema':ab,ti OR 'semaglutide ':ab,ti OR 'Orforglipron':ab,ti OR 'dulaglutide':ab,ti OR 'liraglutide':ab,ti OR 'exenatide ':ab,ti OR 'beinaglutide':ab,ti OR 'Albiglutide':ab,ti OR 'Lixisenatide':ab,ti OR 'loxenatide':ab,ti | 17247 |
|  | #3 | 'randomized controlled trial ':ab,ti OR 'randomized ':ab,ti OR 'placebo ':ab,ti OR 'randomly':ab,ti | 162007 |
|  | #4 | #1 AND #2 AND #3 | 1396 |
| **Cochran Library 1362** | #1 | (obesity ):ab,ti,kw OR (overweight ):ab,ti,kw OR (obese):ab,ti,kw | 59869 |
|  | #2 | (Glucagon Like Peptide 1 Receptor Agonists):ab,ti,kw OR (glucagon-like peptide-1 receptor agonist):ab,ti,kw OR (Retatrutide):ab,ti,kw OR (Tirzepatide):ab,ti,kw OR (Mazdutide):ab,ti,kw OR (CagriSema):ab,ti,kw OR (semaglutide ):ab,ti,kw OR (Orforglipron):ab,ti,kw OR (dulaglutide):ab,ti,kw OR (liraglutide):ab,ti,kw OR (exenatide ):ab,ti,kw OR (beinaglutide):ab,ti,kw OR (Albiglutide):ab,ti,kw OR (Lixisenatide):ab,ti,kw OR (Loxenatide):ab,ti,kw | 5916 |
|  | #3 | (randomized controlled trial ):ab,ti,kw OR (randomized ):ab,ti,kw OR (placebo ):ab,ti,kw OR (randomly):ab,ti,kw | 1379376 |
|  | #4 | #1 AND #2 AND #3 | 1362 |
| **Total** |  |  | 4580 |

**Supplementary Table 2** Other drugs used during the experiment.

| **Study,**  **First author year** | **study drugs** | **Concomitant medications** | **Exception** | **Gluose control medications^a^** |
| --- | --- | --- | --- | --- |
| Louis 2024 | tirzepatide | Permitted used | interfered efficacy and safety of study drugs | Permitted but exception GLP-1RAs and DPP-4 |
| Sean 2023 | orforglipron | Permitted used |  | not permitted |
| Filip 2023 | oral semaglutide | Permitted used | any anti-obesity medication | - |
| Ania 2023 | retatrutide | Permitted used | topical, intraocular, intranasal, injection, or inhaled glucocorticoid etc. | not permitted |
| Domenica 2022 | semaglutide | Permitted used | any anti-obesity medication | - |
| Ania 2022 | tirzepatide | Permitted used | topical, intraocular, intranasal, injection, or inhaled glucocorticoid etc. | not permitted |
| Timothy 2022 | semaglutide | Permitted used | any anti-obesity medication | not permitted |
| John 2021 | semaglutide | Permitted used | any anti-obesity medication | not permitted |
| Thomas 2021 | semaglutide | Permitted used | any anti-obesity medication | not permitted |
| Julie 2021 | liraglutide | Permitted used | - | - |
| Carel 2017 | liraglutide | Permitted used | - | Permitted but exception GLP-1RAs and insulin |
| Xavier 2015 | liraglutide | Permitted used | - | - |

Abbreviations: glucagon-like peptide-1 receptor agonists, GLP-1RAs ; dipeptidyl peptidase 4，DPP-4; ^a^ : patients diagnosed with diabetes during treatment .

**Supplementary Table 3** Results of the Egger test.

| **Aggregate indicators** | **Egger_t** | **Egger P_value** |
| --- | --- | --- |
| Total GLP-1RAs | 2.73 | *0.0134* |
| Liraglutide | 2.05 | *0.1769* |
| Orforglipron | -0.76 | *0.5269* |
| Semaglutide | -0.46 | *0.6884* |
| Retatrutide | 0.28 | *0.8089* |
| Tirzepatide | 0.92 | *0.4560* |

Abbreviations: glucagon-like peptide-1 receptor agonists, GLP-1RAs.


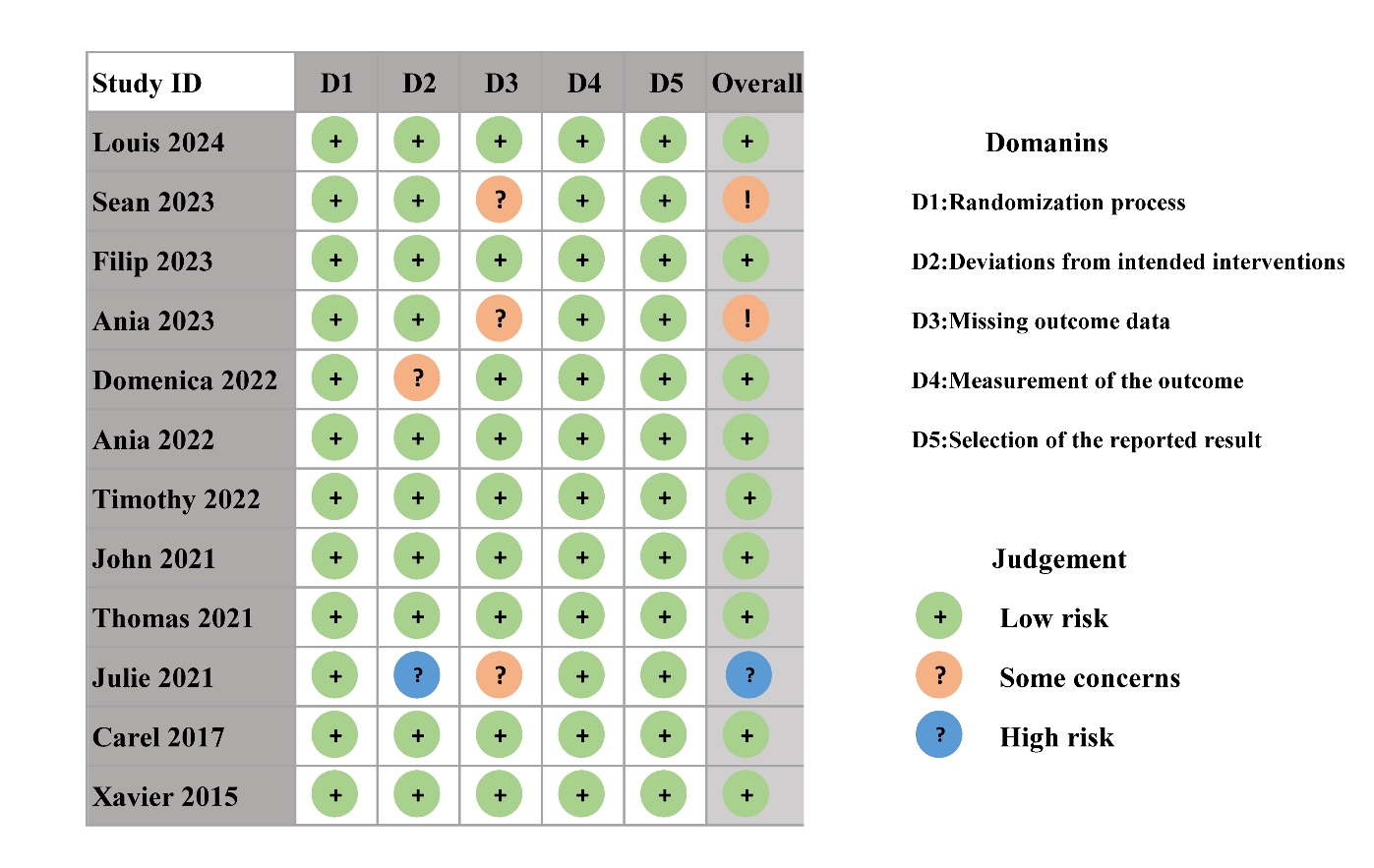


**Supplementary Figure 1** Assessment of the risk of bias in the included studies.


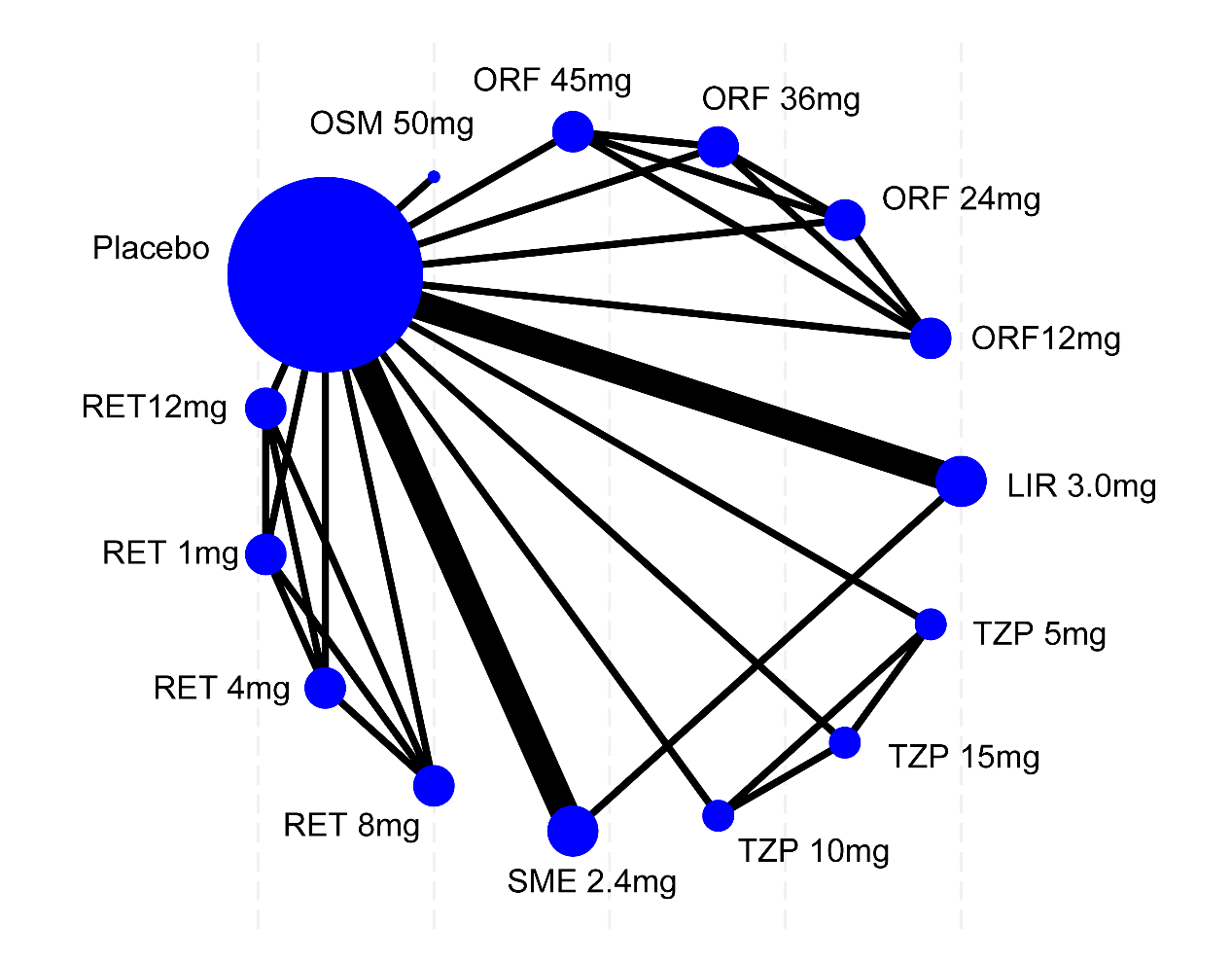


Abbreviations: TZP, tirzepatide; ORF, orforglipron; OSM, oral semaglutide; RET, retatrutide; SME, semaglutide; LIR, liraglutide.
**Supplementary Figure 2** Network plots of included GLP-1 RAs.


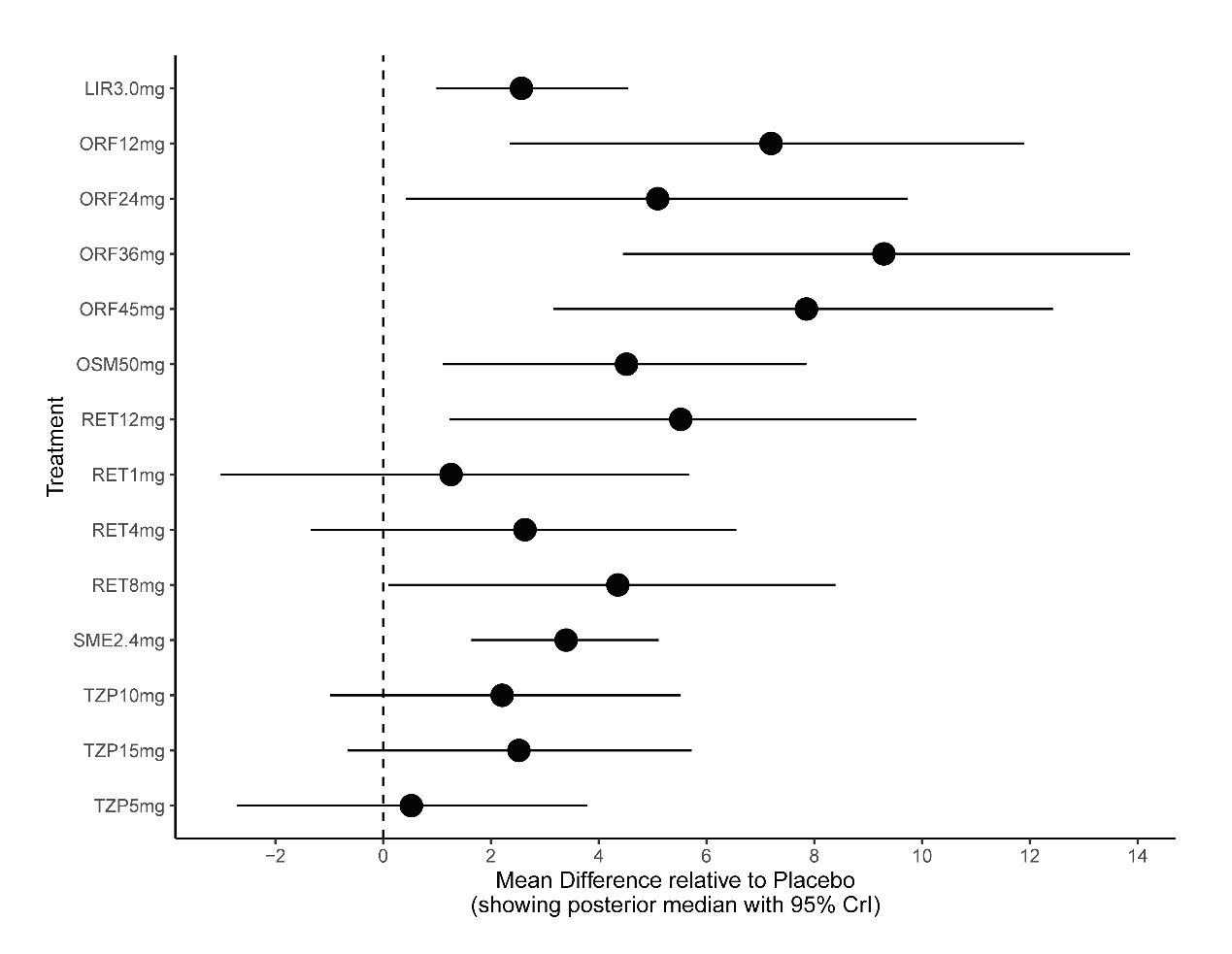


Abbreviations: TZP, tirzepatide; ORF, orforglipron; OSM, oral semaglutide; RET, retatrutide; SME, semaglutide; LIR, liraglutide.
**Supplementary Figure 3** Forest plots of network meta-analysis.


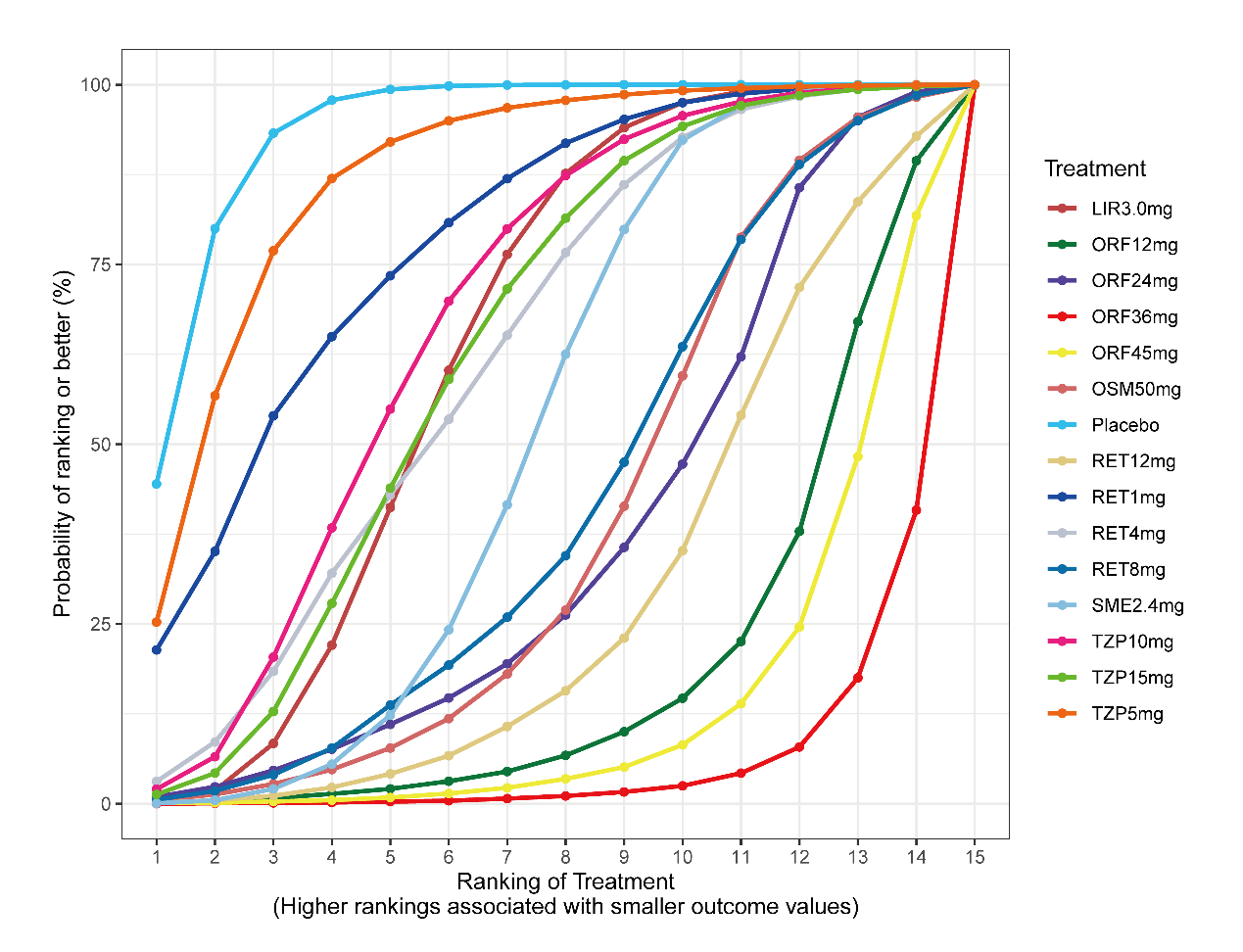


Abbreviations: SUCRA, surface under the cumulative ranking curve; TZP, tirzepatide; ORF, orforglipron; OSM, oral semaglutide; RET, retatrutide; SME, semaglutide; LIR, liraglutide.

**Supplementary Figure 4** SUCRA plots of network meta-analysis.


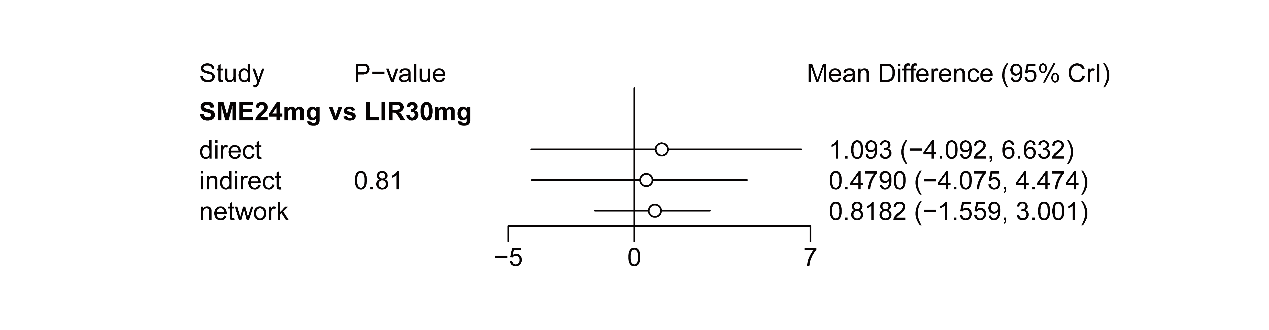


Abbreviations: SME, semaglutide; LIR, liraglutide.
**Supplementary Figure 5** Consistency of network meta-analysis.


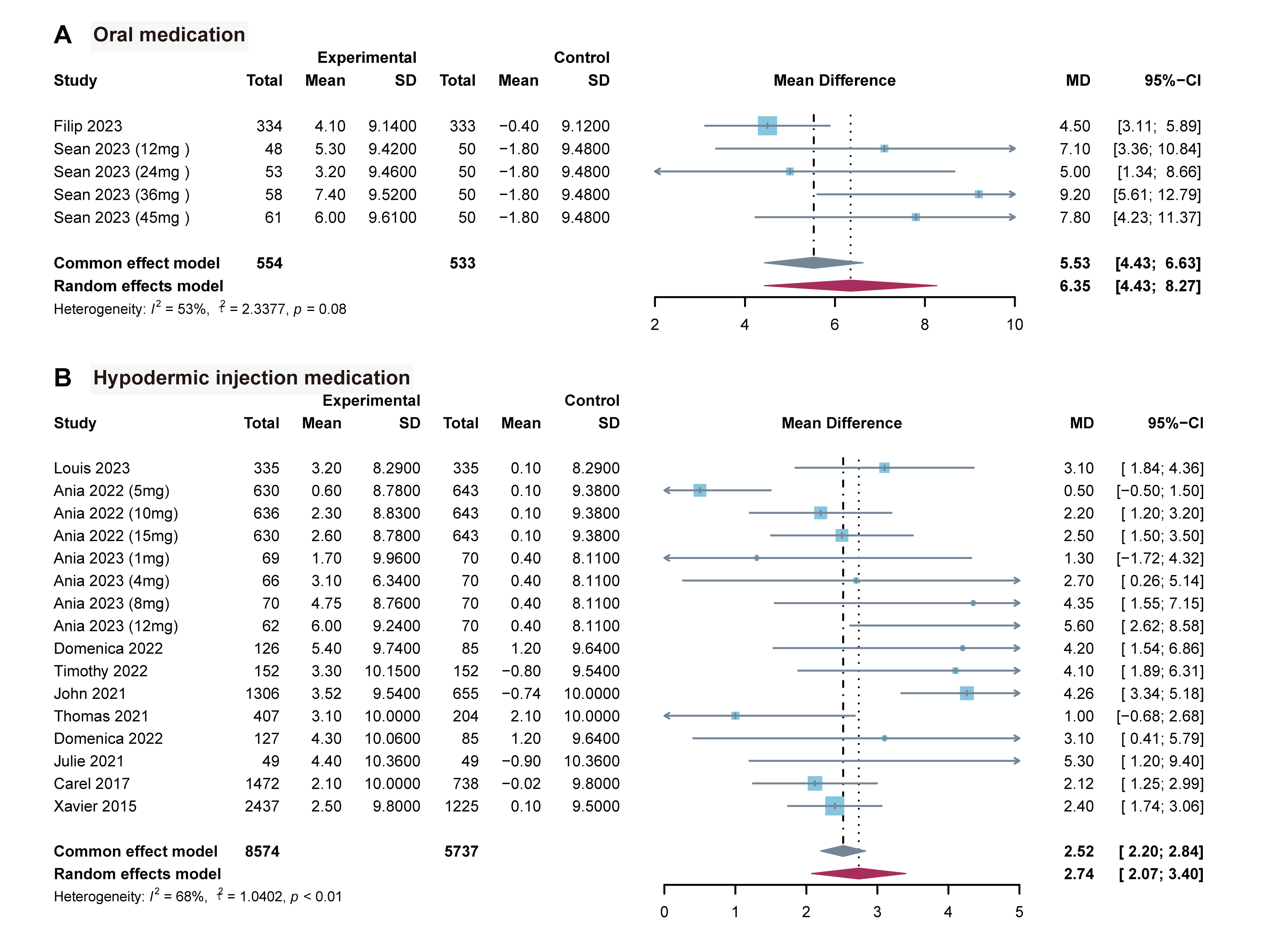


**Supplementary Figure 6** Subgroup analysis of oral or injection medication.


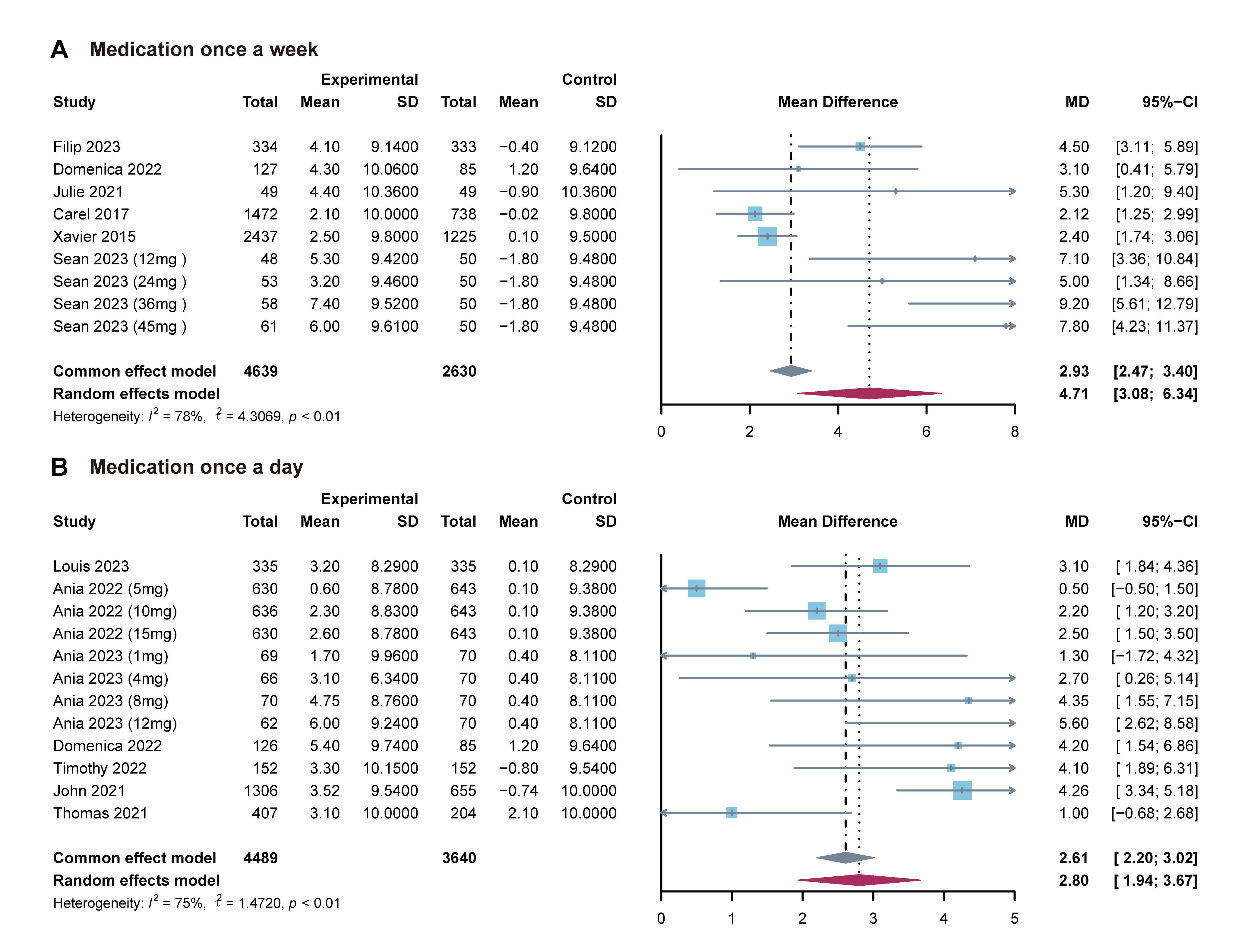


**Supplementary Figure 7** Subgroup analysis of medication interval.


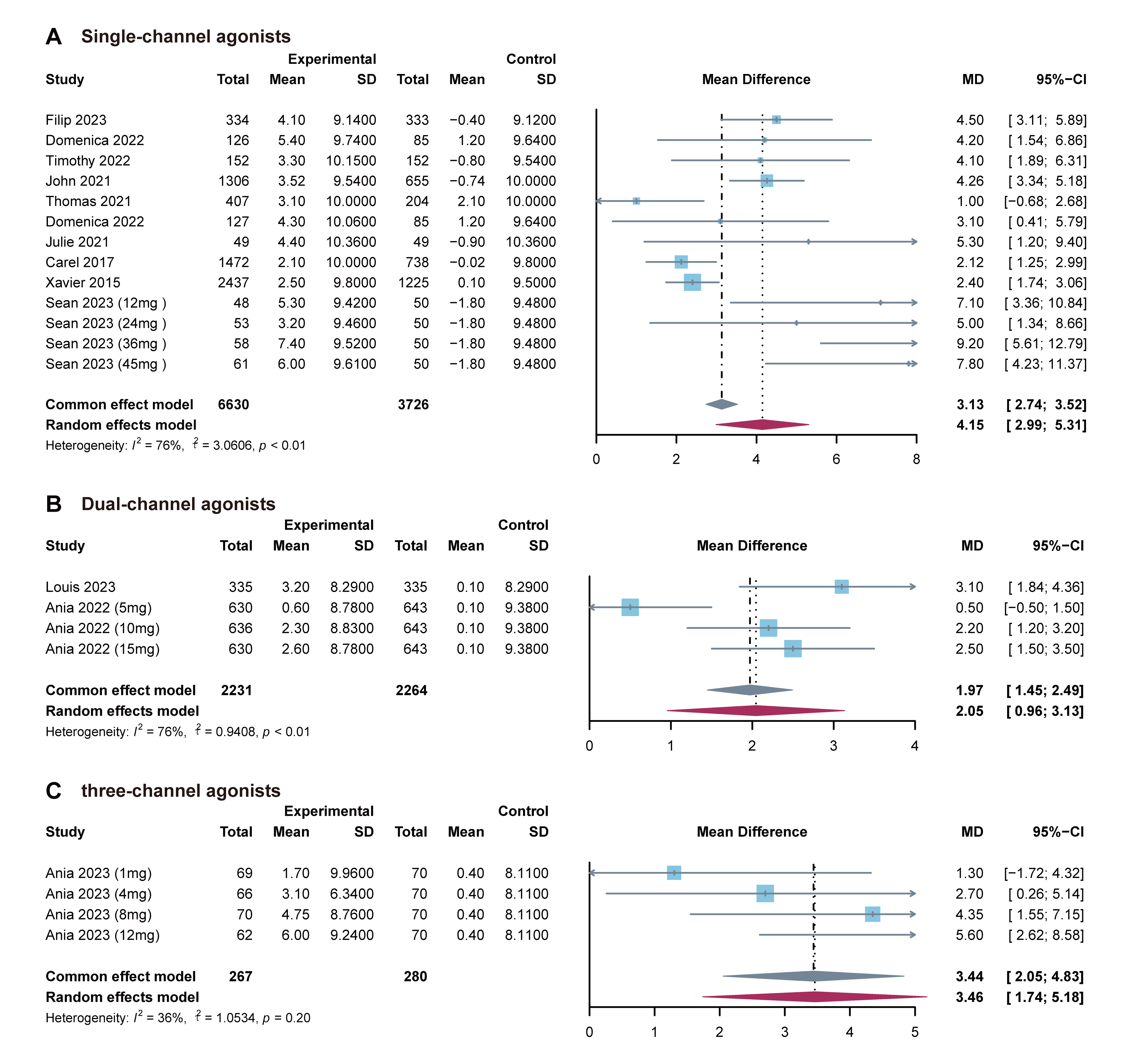

**Supplementary Figure 8** Subgroup analysis of activated channels.


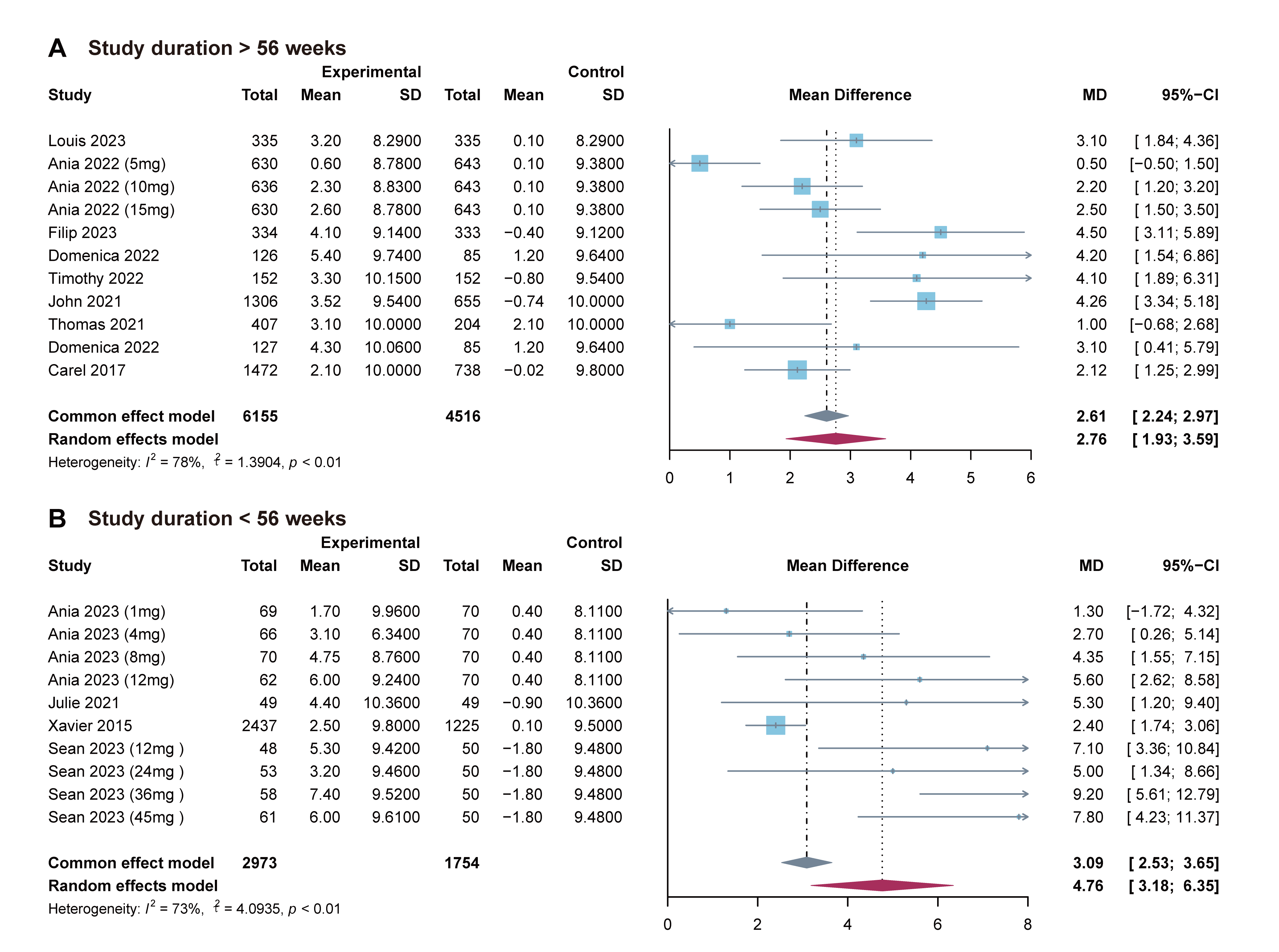


**Supplementary Figure 9** Subgroup analysis of treat time.


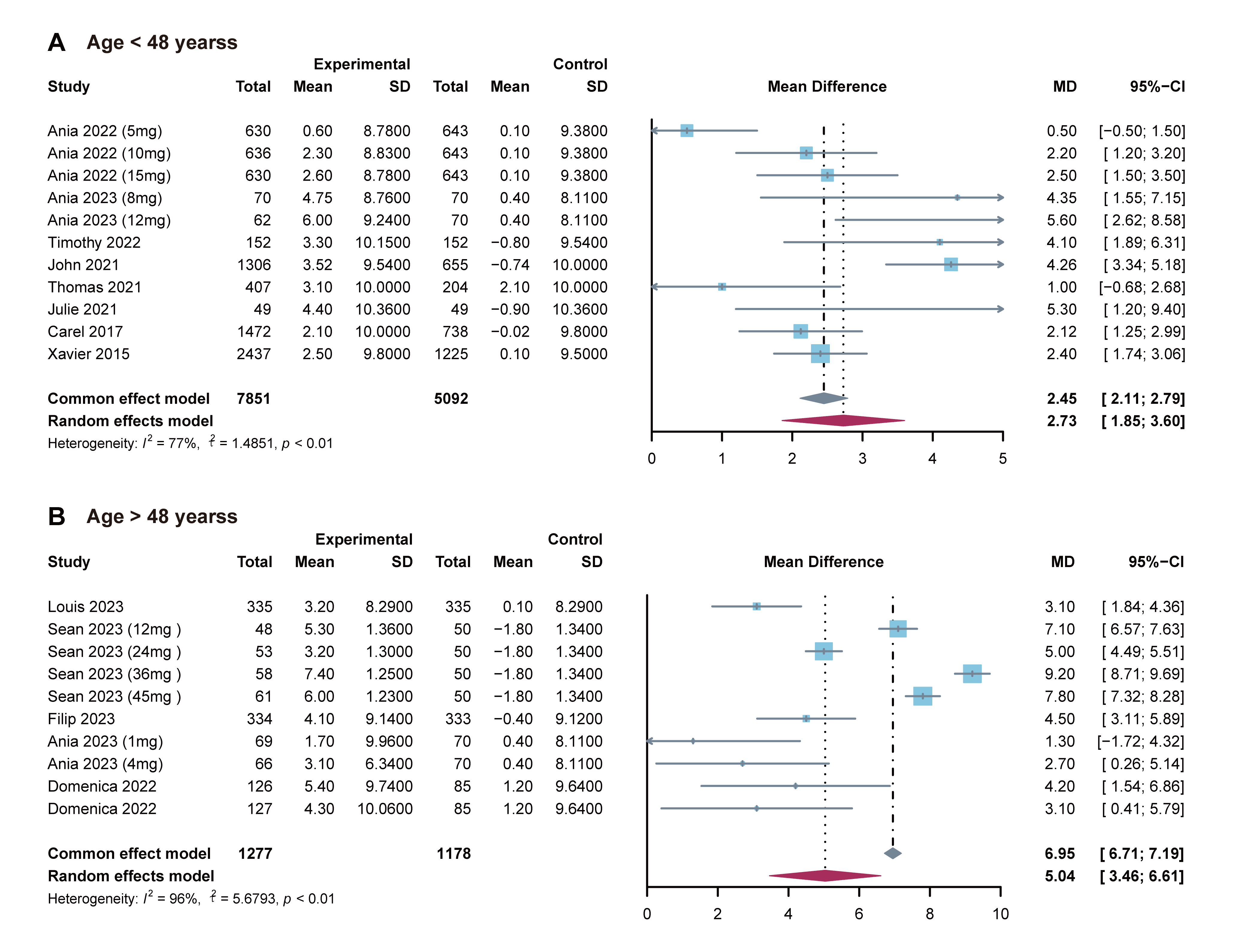


**Supplementary Figure 10** Subgroup analysis of age.


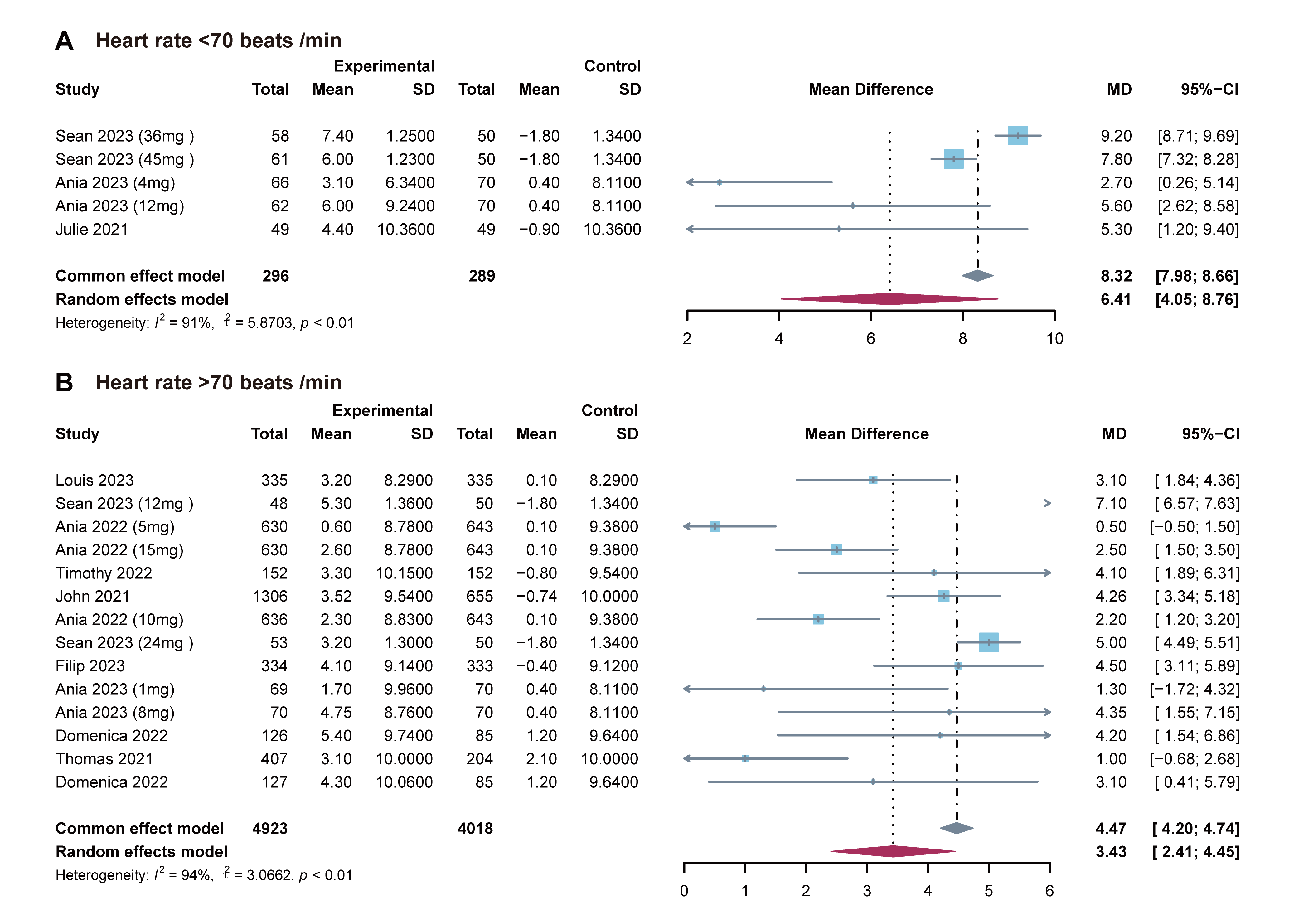

**Supplementary Figure 11** Subgroup analysis of baseline heart rate.


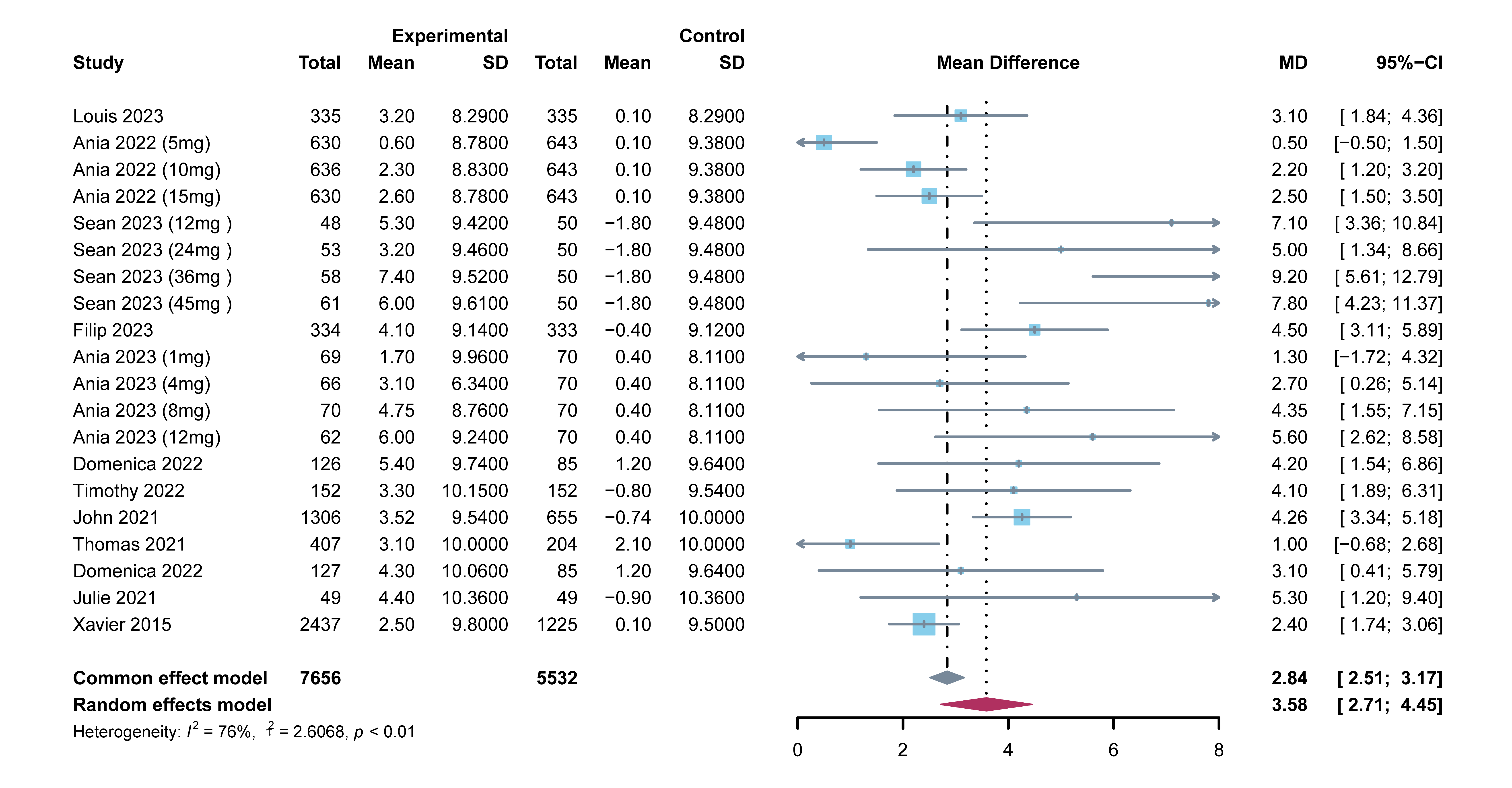

**Supplementary Figure 12** Sensitivity analysis of randomly deleting one study.


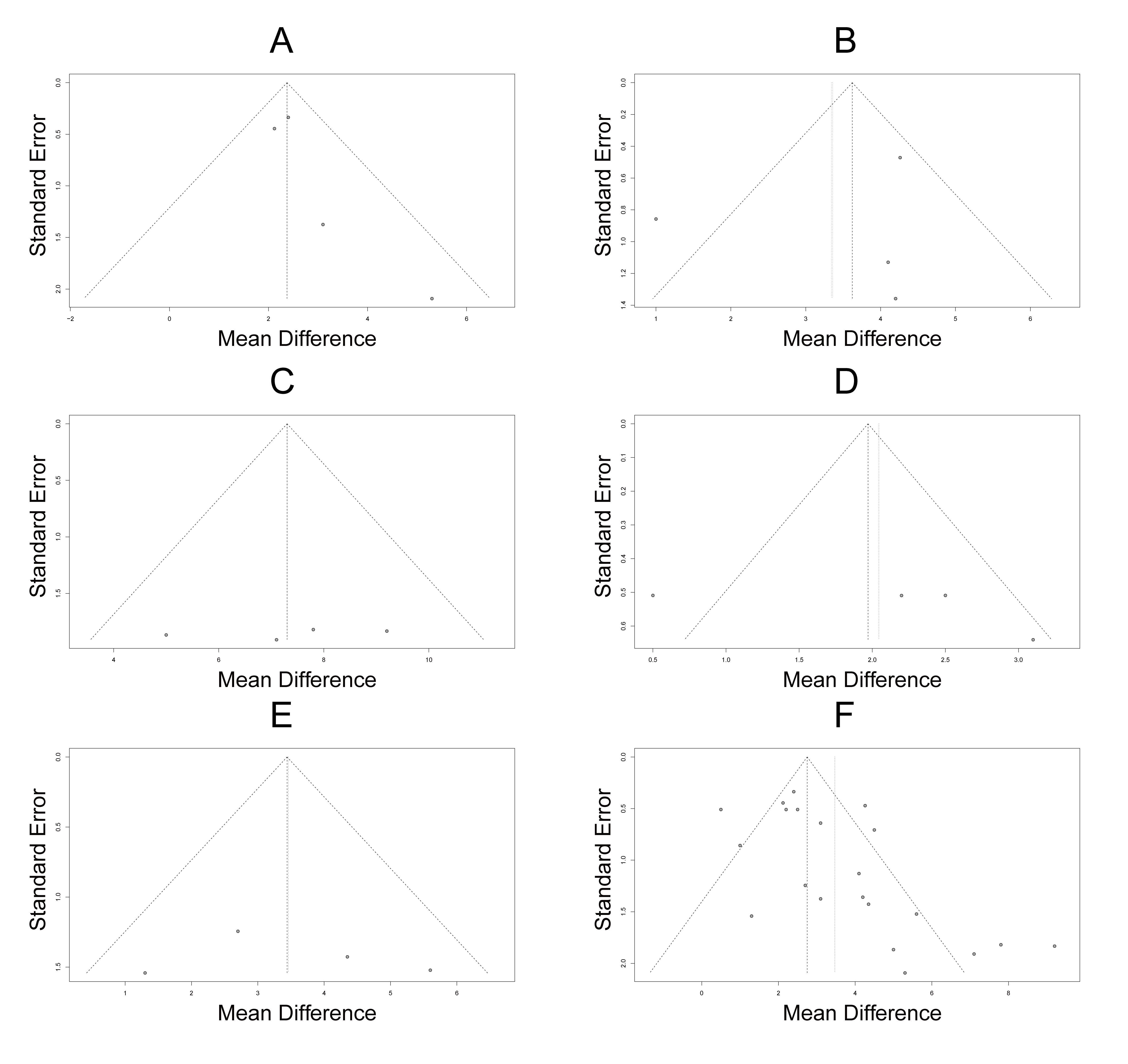


A, liraglutide; B, semaglutide; C, orforglipron; D, tirzepatide; E, retatrutide; F, total GLP-1RAs.
**Supplementary Figure 13** Funnel plots of pairwise meta-analyses.


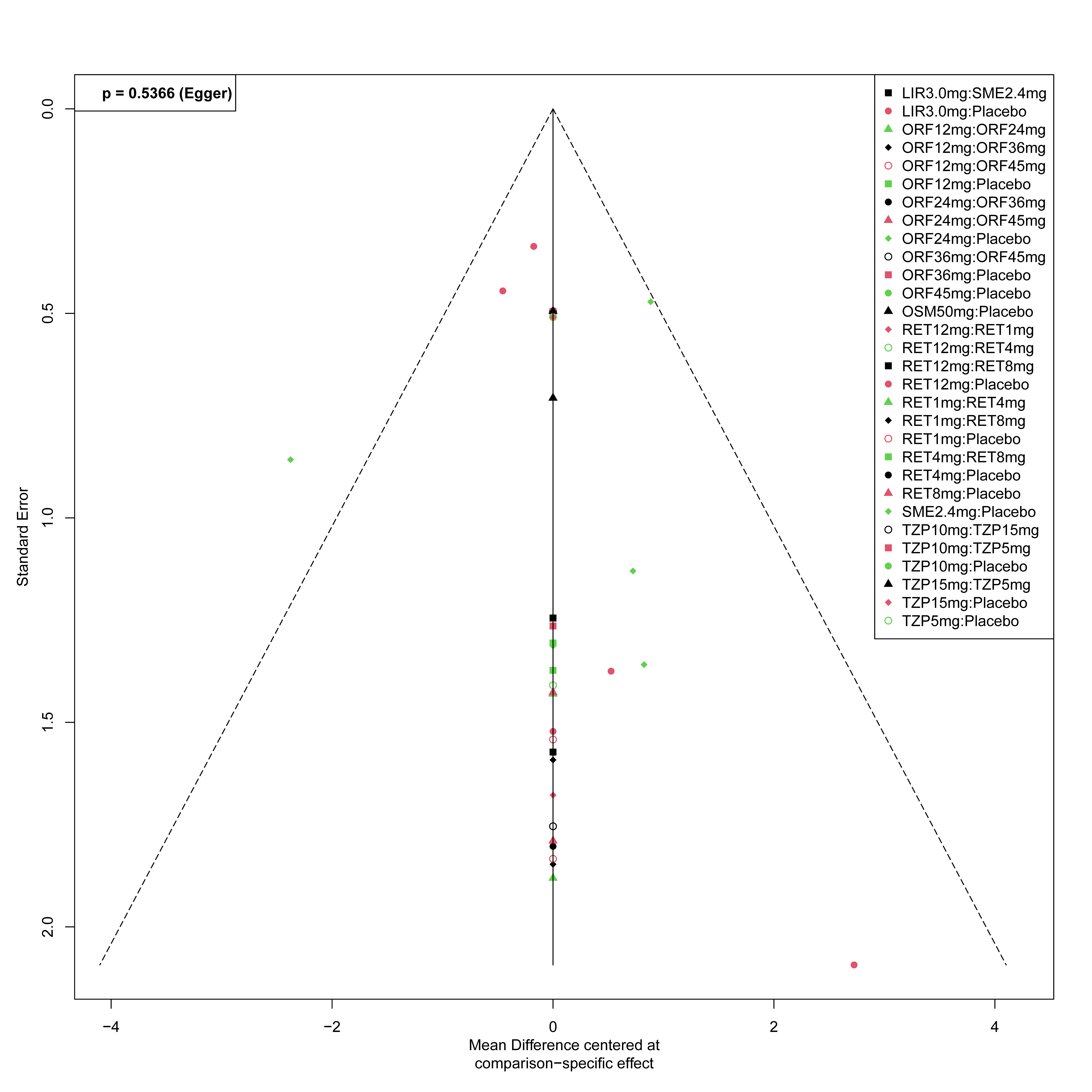


Abbreviations: TZP, tirzepatide; ORF, orforglipron; OSM, oral semaglutide; RET, retatrutide; SME, semaglutide; LIR, liraglutide.

**Supplementary Figure 14** Funnel plots of network meta-analyses.


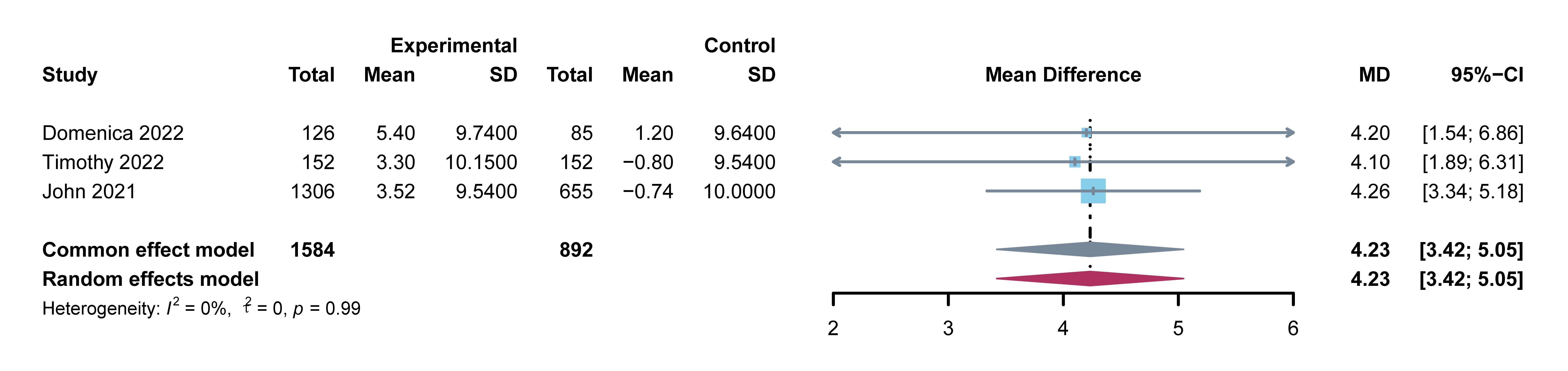


**Supplementary** **Figure 15** forest of semaglutide after excluding one study.


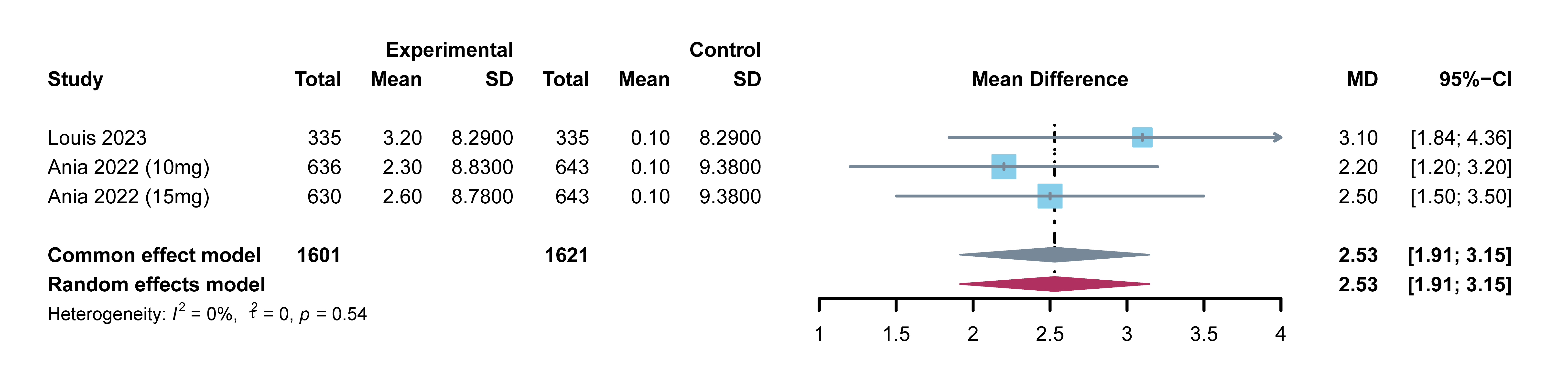


**Supplementary Figure 16** Forest of tirzepatide after excluding one study.
